# Translating clinical trials from human to veterinary oncology and back

**DOI:** 10.1186/s12967-015-0631-9

**Published:** 2015-08-15

**Authors:** Irene Fürdös, Judit Fazekas, Josef Singer, Erika Jensen-Jarolim

**Affiliations:** Department of Comparative Medicine, Messerli Research Institute, University of Veterinary Medicine Vienna, Medical University Vienna and University Vienna, Vienna, Austria; Comparative Immunology and Oncology, Institute of Pathophysiology and Allergy Research, Center of Pathophysiology, Infectiology and Immunology, Medical University of Vienna, Vienna, Austria

**Keywords:** Clinical trials, Human medicine, Veterinary medicine, Dog, Canine, Oncology, Translational

## Abstract

In human medicine clinical trials are legally required for drug development and approval. In contrast, clinical trials in small animal cancer patients are less common and legally perceived as animal experiments. Comparative oncology has been recognized as a method to speed up the development of medications by introducing animal patients with naturally developing tumours. In such cases, using animal patients would generate more robust data, as their spontaneous disease resembles the “real life” situation and thus could be more likely to predict the situation in human disease. This would not only provide veterinary oncology access to the latest developments in medicine before they are available for clinical use in animals, but could also lead to generation of clinical data in animal patients that could be translated to humans. Nevertheless, there are several limitations to practical conduct of clinical trials in veterinary medicine. In this review, the possible application of similar standards of Good Clinical Practice as in human clinical drug development will be discussed in detail, with special consideration of legal and ethical aspects in Europe and the US.

## Background

### Clinical trials in human medicine

In order to receive market authorization of a drug for use in human medicine, all stages of development have to be passed, not only basic research, preclinical and long-term toxicity studies in animal experiments, but also clinical studies of the phases I (testing pharmacology and safety), II (efficacy, safety, dose finding) and III (therapeutic confirmatory trials in larger patient numbers) [1]. Phase I studies are usually conducted in healthy volunteers (probands), except in oncology where patients are already involved due to higher risk of toxicity of substances [2].

Whenever the primary purpose of using a medicinal product in humans is to gain scientific knowledge, this must be defined as a clinical trial. For the patient, the difference to normal medical treatment is that the therapy is predefined in a study protocol. There must be as little deviation from this study plan as possible to assure data quality, which in return means that the individual patient’s needs are not the first focus of the treatment [3]. As of course the safety of the patients has to be ensured, as well as the quality in the development of medical products, the conduct of clinical trials is strictly legally defined.

#### Legal requirements (overview)

Large Phase III and IV studies are usually conducted as international multicentre studies. Therefore, harmonisation of laws and directives is necessary on an international level. The international guidelines for Good Clinical Practice (GCP) have been harmonised between Europe, the USA and Japan within the “International Conference on Harmonisation” (ICH). The result is the ICH-GCP guidelines that define the international standard for the conduct of clinical trials in human medicine [4]. These guidelines are very detailed and in some aspects they even go further than national laws. Hence, they can be followed without difficulties in interpretation [5]. Still, they only serve as a “note for guidance” and therefore are considered recommendations according to the state of knowledge and not binding law. However, several elements of ICH-GCP have been implemented in the European Directive 2001/20/EC [6] and national laws (e.g. the Austrian “Arzneimittelgesetz” [7]) as well as in the US regulations relating to GCP and clinical trials (Code of Federal Regulations, Title 21 [8]). Table 1 gives an overview of laws and guidelines for Good Clinical Practice, applicable for clinical trials conducted in EU member states and corresponding regulations applicable in the USA.

**Table 1:** Overview of GCP-regulations valid for EU-member states and the USA

Country	Regulation	Legal status
EU	EU-GCP “Directive 2001/20/EC” [6]	Binding law
EU	Guidance documents of the European Commission (i.e. European Commission EUDRACT 2004 [9], European Commission Eudravigilance 2004 [10])	Guidance on the implementation of requirements in Directive 2001/20/EC
EU (individual member states)	National laws of EU member states (e.g. the Austrian “Arzneimittelgesetz von 1983, Fassung 2013” [7])	National laws, implementing Directive 2001/20/EC
EU and USA	ICH-GCP-guidelines [4]	Recommendations, aiming for harmonisation of GCP between USA, Europe and Japan
USA	Code of Federal Regulations (CFR Title 21) [8]	Binding laws and regulations
USA	FDA GCP/Clinical Trial Guidance Documents [11]	Guidance on the implementation of GCP-regulations

The key elements of GCP are a description of duties and the assignment of these to the different parties responsible for conducting the clinical trial. Those parties are the sponsor of the trial (an organisation or a pharmaceutical company, responsible for the initiation, realization and funding), the investigator (a physician, responsible for the trial at the study site) and the monitor (a person assigned by the sponsor who assures data quality by regularly visiting the study site) [12]. Furthermore, the role of ethics committees, including their responsibilities in respect to patient informed consent and safety information of the investigational product, is depicted. One further aspect regarding GCP is the documentation of the trial, including standard operating procedures (SOPs) provided by the sponsor, audits in the form of independent quality control, and archiving of study documents [12].

The first step before any clinical trial can start is the submission and review of each study plan by the responsible ethics committee(s) as well as regulatory authorities [7]. Furthermore, each clinical trial has to be registered in a publicly available database (e.g. http://www.clinicaltrials.gov) and an official number has to be assigned (“European clinical trials database EUDRACT” [9]).

The ethics committee and regulatory authorities have to evaluate the study plan and only after their approval can a trial be initiated at a study site. Usually, each institution and/or political district in Austria has its own ethics committee. Nevertheless, according to Article 7 of the EU-Directive 2001/20/EC, one “lead” ethics committee must be assigned to approve multicentre trials with a single opinion for each Member State [6]. Ethics committees lay major emphasis on the patient informed consent procedure, which is often the reason for queries and adaptation before a trial is approved. The informed consent form should not only contain all information relevant to the trial, but also objectively explain all risks and benefits to the patient to enable him/her to freely consent to participate in the clinical trial. The critical point here is finding a balance which provides patients with complete information while keeping the explanation simple enough to understand. If patients are not able to consent (e.g. mentally disabled people or children), their legal representative has to take over this responsibility and decide in the patients’ best interest [6].

Another ethically relevant aspect with respect to patient security, which is decisive to whether a clinical trial can be continued, is the regular review of safety-related incidences. This is especially important in early clinical trials (Phase I and II), where investigational products are tested for which the potential toxicity to humans has not been adequately studied. The investigator is therefore required to report any serious adverse event (SAE) that occurs in a study patient within 24 h to the sponsor of the trial [7], who reviews and evaluates whether there is a causal relationship between the event and the study drug. If this is the case, it must be reported as “suspected unexpected serious adverse reaction” (SUSAR) to regular authorities, ethics committees, and all other participating study sites according to the Eudravigilance guidelines of the EU [10] or the FDA’s “Guidance on Adverse Event Reporting” [13].

#### Sponsorship (the role of pharmaceutical industry)

Sponsorship according to ICH-GCP [4] is *“*an individual, company, institution, or organization which takes responsibility for the initiation, management, and/or financing of a clinical trial” (p.10). As depicted in that definition, the financier does not necessarily have to be responsible for conducting the trial, nor does the party conducting the trial have to provide finance. Clinical trials can be run as academic research, funded by e.g. federal programs or charity organizations. However, clinical drug development is not only subject to unprecedented regulatory pressures, but it is also very costly [14]. This is especially the case in oncologic indications, leading to the fact that in practice it is almost impossible to fund a clinical trial program without involvement of the pharmaceutical industry. This hypothesis is supported by a recent study on clinical trials in asthma, where in 95 % of included clinical trials involvement of the pharmaceutical industry was reported [15].

Of course companies mainly support trials that fit into their own drug development program for a given indication [16], so they review carefully what and how much they will support. Generally, such collaboration between academia and the pharmaceutical industry can be beneficial for both parties. The academic sector gains access to financial resources and technology, while the pharmaceutical industry acquires the clinical expertise and capabilities on which it is dependent [14]. Nevertheless, criticism with respect to the almost unavoidable involvement of pharmaceutical companies might be raised because of (1) potential biases in study results, (2) the possibility of non-reporting of negative results, or (3) non-objective interpretation of results in order to fit the company’s interests [17]. One further critical point in this respect is transparency of payments, and that funding of scientific research has to be clearly separated from prescription of a drug. Otherwise clinical data may come under suspicion of being influenced by financial interests. In order to avoid this, the Food and Drug Administration (FDA, federal agency of the United States responsible for drug approval) always requires financial disclosure from investigators as part of registration trials [18].

In conclusion, the pharmaceutical industry plays a major role in bringing clinical trial programs into practice, not only from a financial perspective but also with respect to ICH-GCP compliant implementation of studies. Nevertheless, it has to be critically considered how much investigators are connected to and/or dependent on the pharmaceutical industry, being aware of potential biases in setup and results of studies that have been initiated by companies, favouring their own drugs.

### Clinical trials in veterinary medicine

In veterinary medicine the status of clinical trials is quite different (Table 2). Furthermore, they are not nearly as common as in human medicine. This is specifically apparent in oncology, an area where development of new treatment strategies is urgently needed. The Veterinary Cancer Society’s database “vetcancertrials.org” [19] revealed a total of 121 trials (worldwide, all tumour types) in animal patients, whereas a similar search performed at “clinicaltrials.gov” resulted in 18.387 studies in human patients with cancer and other neoplasms [20].

One reason for this difference is that, according to EU-regulations, in veterinary medicine a drug can be approved without additional clinical trials in animal patients if sufficient evidence of efficacy and safety is given for humans [21]. In the USA, clinical trials in animal subjects are implemented in laws on veterinary drug approval, however, focussed on the investigational product [22]. Furthermore, the conduct of GCP-compliant veterinary clinical trials, as opposed to human clinical trials, is not clearly defined by law, even though they are almost equally time- and cost-intensive. In the following, the legal and ethical presuppositions and differences are depicted and compared to the most up-to-date information on human clinical trials.

**Table 2:** Overview of GCP-regulations for clinical trials in veterinary medicine valid for EU-member states and the USA

Country	Regulation	Legal status
EU (individual member states)	National laws of EU member states (e.g. the Austrian “Tierversuchsgesetz 2012” [23])	National laws (e.g. Austria:, clinical trials perceived as animal experiments)
EU	Directive 2010/63/EU on protection of animals used for scientific purposes [24]	Not applicable for clinical trials in animals
EU and USA	VICH Consensus Guidelines for Good Clinical Practice [25]	Recommendation
USA	Good laboratory practice for nonclinical laboratory studies (CFR Title 21, Part 58 [26])	Not applicable for clinical trials in animals
USA	Animal Welfare Act and Animal Welfare Regulations [27]	Applicable for animals used for scientific purposes

#### Legal requirements

According to the EU-Directive 2001/82/EC [21] for the development of new veterinary medical products, pre-clinical tests as well as clinical trials are required in order to fulfil premises related to safety and efficacy, respectively. For market authorisation of a new veterinary medical product these are required as well, but do not need to be—in contrast to human medicine—solely based on data generated in clinical trials. Moreover, if a product or its active substances have been in “well-established veterinary use within the Community for at least ten years, with recognised efficacy and an acceptable level of safety”, no tests or clinical trials are required to apply for an authorisation if “appropriate scientific literature” is provided [28]. This gives an impression of how broadly the regulation can be interpreted, and shows that there are alternatives to cost- and time-intensive clinical trials if a company chooses to apply for registration of drugs for veterinary use. Similarly in the USA the approval of a new drug is possible without clinical testing in animals if “omitted as related to laboratory studies and prior clinical experience” [22].

According to the Austrian “Tierversuchsgesetz” [23], clinical trials in animal patients have to be managed like animal experiments concerning the involvement of and approval by an ethical review board and regulatory authorities. Austrian law in this respect is stricter than the European Directive on the protection of animals used for scientific purposes, in which it is even pointed out that this Directive is not applicable for clinical trials in animals [24]. In the USA, minimal criteria for humane care and use of animals in research are topic of the Animal Welfare Act [27]. Animal clinical research is not completely covered by federal rules and has to be supported by strong institutional policies in case where there are no other rules [29].

However, guidelines exist (although not enforceable by law) aiming to harmonise design and conduct of clinical trials with veterinary products in EU member states but also in the USA: the Consensus Guideline for Good Clinical Practice published by the European Agency for the Evaluation of Medicinal Products (EMA) and FDA [25]). This guideline mainly focuses on ensuring data quality for market authorisation of a veterinary medicinal product. It states that “this guideline should be followed when developing clinical study data that are intended to be submitted to regulatory authorities.” (p.2), but also “An alternative approach may be used if such an approach satisfies the applicable regulatory requirements.” (p.27) [25].

Although according to EU-Directive 2001/82/EC [21] clinical trials are at least recommended in veterinary medicine, they are still not obligatory like in human medicine, and no regulations or standards exist that could be legally enforced. Still, progress is being made, and a first step is the CVMP/VICH guidelines [25] that try to set standards on implementation of veterinary clinical trials and other attempts. Additionally, veterinary study groups have been formed that publish guidelines (i.e. “Guiding the Optimal Translation of New Cancer Treatments From Canine to Human Cancer Patients”, published by Khanna et al. [30]).

#### Only an animal experiment? Differences to a clinical trial in veterinary patients

As depicted above, almost no binding regulations exist specifically for conducting clinical trials in veterinary medicine. The regulations for animal experimentation are applied to this setting as well, and thus every experimental use of animals that implies any burden for the animal has to be reviewed and approved by an ethical review board and regulatory authorities [23]. Even though this sounds quite similar to the processes in human clinical trials, the criteria that the application must meet are less strictly defined. Furthermore, as the application to the ethics committee has to be done in accordance with regulations regarding lab animals, some points are not applicable (e.g. housing of pet dogs can’t be standardised or assured to meet the criteria for lab animals) and some important aspects are not mentioned at all (i.e. predefined quality standards for the owner informed consent form).

Regarding the informed consent procedure, there is also significant variation concerning how much information the owner receives [29]. This can range from a general statement regarding potential implications to the animal (as required in institutional guidelines [31]), to a detailed description of side effects and potential risks and benefits to provide the owner with all information necessary to decide in representation of his or her animal. Furthermore, some other standard requirements of the ethics committee in human clinical trials are not applicable in the veterinary setting, like continuous safety reporting and evaluation/monitoring by the sponsor during the study or annual prolongation of the approval.

However, using animal patients with naturally occurring diseases would be beneficial in generating more robust data, as disease development is more likely to mimic the “real life” situation, and thus could be more likely to predict the situation in human disease. The animal patient has the potential to serve as a “clinical animal model” for human disease, due to striking similarities and homologies in diseases, as observed in e.g. the role of HER-2 (human epidermal growth factor receptor-2) in breast cancer [32]. In this case the benefit is not only on the human but also on the animal side, as the animal patient could have access to the latest developments in medicine before they are available for clinical use. Moreover, this could also relieve some of the burden on lab animals.

In contrast to animal experimentation, the conduct of clinical trials in animal patients would be one approach to reduce laboratory animal experiments and to bring substances into clinical medicine earlier. This would also address the “3Rs” (reduction, replacement, refinement) as a key concept in order to reduce and limit the amount of unnecessary pain, suffering and distress for laboratory animals [33]. Lab animal models for oncologic indications are artificially bred to develop cancer, resulting in severe, long lasting burden connected to the tumour disease and ending in sacrificing them after the experiment [23].

**Fig. 1:**
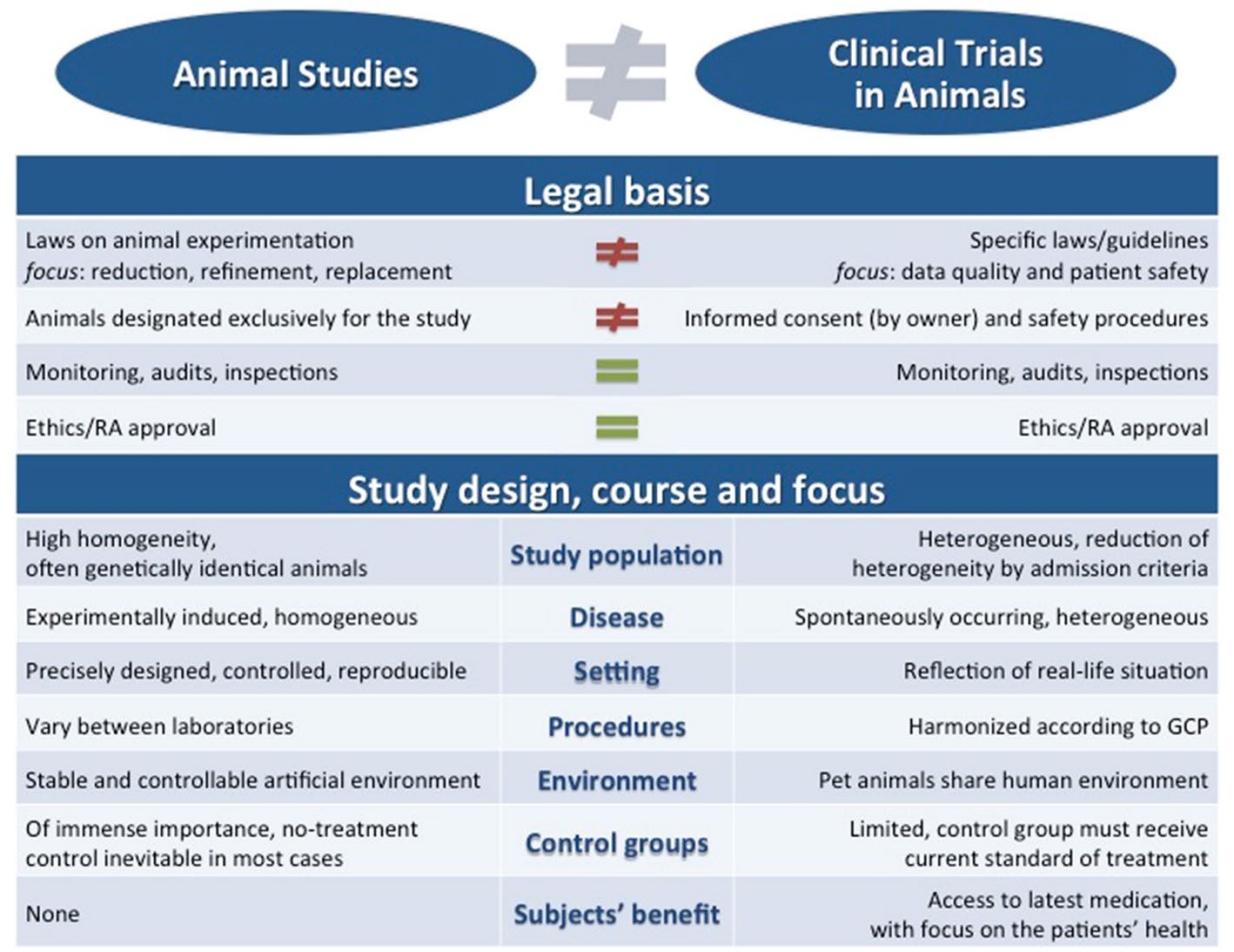
Overview on the differences between animal experiments and clinical trials in a legal sense but also regarding their scientific aims and the role of the animals used

An animal experiment which tests a new drug is done to evaluate its mode of action, efficacy and also safety. However, its limitations are the laboratory settings, often inbred strains, or using an “artificial” disease in an otherwise healthy animal. Furthermore, animal experiments are not able to mirror the human tumour growth over a longer period of time, or the characteristics of minimum residual disease, as well as the heterogeneous macro- and microenvironment as in spontaneously occurring human cancer [34] (Fig. 1).

Accordingly, in clinical development it has been observed that much data generated in mouse or other animal models is not reproducible in humans [35]. Thus, the dog patient might be able to assist in the transition between laboratory research on mouse models and clinical trials in human patients [34].

#### Sponsorship (the role of pharmaceutical industry)

According to the EU-Directive 2004/28/EC (9) [21]: “The costs of research and development to meet increased requirements as regards the quality, safety and efficacy of veterinary medicinal products are leading to a gradual reduction in the range of products authorised for the species and indications representing smaller market sectors.”

Taking this statement into consideration, the availability of a drug does not seem based on its clinical efficacy, but more on the strategic interest of the pharmaceutical industry. As discussed for clinical trials in humans, companies base their decision for or against the development of a new drug not only on its possible clinical efficacy (that can differ between in vitro results and clinical data as stated above), but first and foremost on economic considerations and the potential competition with their own authorized products. The more seldom a disease occurs or the less patients available to treat, the less those investments for data generation can be financed and the less profit a company acquires after market authorisation. This implicates a kind of “bottle-neck effect” in available clinical data: only the most profitable will get through. The EU-Directive tries to address this point in Article 18: “There is also a need to stimulate the interest of the veterinary pharmaceuticals industry in certain market segments in order to encourage the development of new veterinary medicinal products.” [21].

Veterinary indications thus are not the most interesting segments for pharmaceutical industry, as not only the number of patients that could possibly be treated in an indication is lower than in comparison to human medicine, but also the situation regarding payment of therapies is different. In the veterinary setting generally there is no healthcare structure with compulsory insurance, as is common for humans, so animal owners have to pay therapy costs out of pocket. This in addition is accompanied by the possibility of euthanizing an animal with a non-curable disease like cancer, which might facilitate an owner’s decision not to further invest in therapies.

On the other hand academics, and hence research being independent from the interests of the pharmaceutical industry, play an important role, not only in basic but also in clinical research. To align with GCP and all regulatory and ethical requirements also means that financial investment and funding by third parties is necessary. The central funding organisation for basic research, including animal experimentation, in Austria is the Austrian Science Fund (FWF), but there are also others, i.e. charity associations and foundations. If one intends to apply for financial support for a clinical trial in a veterinary indication at those institutions, the first difficulty is that there is no applicable category for animal clinical trials. Moreover, funding agencies constantly reply that a pharmaceutical company should be approached first or would usually be the right contact for these kinds of requests. So in this respect as well, clinical trials in animals have an exceptional position that has been unsatisfactorily addressed up to now, with the rare exception of private foundations as the National Canine Cancer Foundation (NCCF) in the USA [36].

## Conclusion

Clinical trials in veterinary medicine are unfortunately not performed as routinely as in human medicine and commonly involve only small patient cohorts. This might be (1) due to the lack of laws that require and define the conduct of clinical trials in veterinary medicine (not just in the form of guidelines, but legally binding), and (2) because other cheaper, faster and legally accepted ways to approve drugs for veterinary use do exist (e.g. conducting an (clinical) animal experiment still serves the purpose of drug-approval). Furthermore, if no veterinary substance is available for treatment of companion animals, legislation [37] gives permission to use human medications off-label under defined circumstances. This less defined situation with respect to clinical trials in veterinary medicine is in contrast to the strictly defined and legally binding regulations for human clinical trials. Moreover, the focus is different: whereas in human clinical trials regulations are focusing on human protection (in respect to ethics and health), the first scope of the existing animal clinical trial regulations is drug development and safety. Animal welfare and protection is topic of animal welfare legislation, which usually does not specifically cover clinical trials.

In contrast to human clinical research, there have been almost no funding opportunities for veterinary clinical studies. The rare veterinary clinical trials that were conducted according to human GCP-standards have been initiated by well-known pharmaceutical companies (e.g. development of toceranib in mast cell tumours in dogs [38]).

Despite implementation of more specific regulations of clinical trials in animal patients it would be favourable, if (especially in academia) more clinical animal studies would be voluntarily carried out as GCP-compliant clinical trials in animal patients with naturally developed disease. This would not only be of benefit for lab animals as well as animal patients, but also is of translational significance to encourage simultaneous and thus faster development of new drugs [39]. This review concentrating on the situations in Europe and in the US will hopefully stimulate the international discussion on the constant improvement of the legal base for comparative trials.

## Abbreviations

EMA: European Agency for the Evaluation of Medicinal Products
EU: European Union
FDA: Food and Drug Administration
FWF: Austrian Science Fund
GCP: Good Clinical Practice
HER-2: human epidermal growth factor receptor-2
ICH: International Conference on Harmonisation
NCCF: National Canine Cancer Foundation
RA: regulatory authorities
SAE: serious adverse event
SOP: standard operating procedure
SUSAR: suspected unexpected serious adverse reaction
USA: United States of America
